# Effects of a shared decision‐making implementation programme on patient‐centred communication in oncology—Secondary analysis of a randomised controlled trial

**DOI:** 10.1111/hex.14030

**Published:** 2024-03-28

**Authors:** Anja Lindig, Lotta Mannagottera, Pola Hahlweg, Hannah Sigl, Anne Klimesch, Stefan Zeh, Levente Kriston, Isabelle Scholl

**Affiliations:** ^1^ Department of Medical Psychology University Medical Center Hamburg‐Eppendorf Hamburg Germany; ^2^ Department of Psychiatry and Psychotherapy University Medical Center Hamburg‐Eppendorf Hamburg Germany

**Keywords:** Four Habits Coding Scheme, implementation, oncology, patient‐centred communication, randomised controlled trial, shared decision‐making

## Abstract

**Background:**

There is a need for better implementation of patient‐centred (PC) communication and shared decision‐making (SDM) in routine cancer care.

**Objective:**

The aim of this study was to assess whether a programme to implement SDM in oncology had effects on PC communication in clinical encounters.

**Design:**

This study constitutes a secondary analysis of data derived from an implementation trial applying a stepped wedge design that, among other strategies, incorporated training and coaching to enhance the PC communication skills of physicians.

**Setting and Participants:**

We analysed audio recordings of clinical encounters collected in three departments of a comprehensive cancer centre in Germany before and after rolling out the implementation programme.

**Main Variables Studied:**

We assessed the PC communication skills of physicians.

**Main Outcome Measures:**

Each recording was rated by two researchers using the German version of the Four Habits Coding Scheme (4HCS), an observer‐based measure of PC communication. Interrater reliability of the outcome measure was acceptable but moderate. Demographic data of patients participating in audio recordings were analysed.

**Methods:**

Data were analysed using descriptive statistics and linear mixed‐effects models.

**Results:**

In total, 146 encounters, 74 before and 72 after implementation, were evaluated. The mean age of patients was 57.1 years (SD = 13.8), 70.3% were female, the largest portion of patients had medium formal education (32.4%) and were (self‐) employed (37.8%). No statistically significant effect of the implementation programme on the physicians' PC communication skills was found.

**Discussion:**

The results indicate that the investigated programme to implement SDM in oncology, including training and coaching, had no effects on PC communication in clinical encounters. These results are in contrast to other studies that report the effects of specific training or coaching on PC communication. Reasons for the lack of effect include the short duration of our training compared to other studies, limited reliability and moderate interrater reliability of the 4HCS scale, limited reach of the intervention programme as well as the inclusion of physicians regardless of their exposure to the interventions.

**Conclusion:**

Further research is needed to develop implementation strategies that improve physicians' PC communication skills.

**Patient Contribution:**

Data on patients and clinical encounters with patients and physicians were analysed. There was no other patient or public involvement.

## 1 INTRODUCTION

One of the key components of German healthcare is patient‐centred care (PCC), which focuses on the values, preferences and needs of a patient.^1^, ^2^ A well‐evaluated patient‐centred (PC) communication concept is shared decision‐making (SDM).^3^ The concept of SDM involves that healthcare professionals (HCPs) and patients participate equally during discussions on healthcare decisions.^4^, ^5^ Accordingly, HCPs explain the risks and benefits of diagnostic or therapeutic methods based on their clinical expertise and evidence‐based knowledge and provide understandable patient information.^6^, ^7^ Patients inform HCPs about their preferences, values and needs.^6^, ^7^ PCC and SDM are crucial in cancer care, where treatment decisions significantly impact patients' quality of life.^2^, ^8^ Consequently, the guideline of the American Society of Communication in Oncology includes aspects of PCC and SDM as necessary for high‐quality communication in oncology (e.g., trustful behaviour of clinician, collaboration, information on benefits and burdens of a treatment).^3^ PC communication skills are prerequisites for SDM adoption in clinical encounters and have positive effects on patient outcomes.^3^, ^9^, ^10^ These skills include, for example, empathy, active listening as well as exploration of the patient perspective.^11^

Existing studies report that PC communication of HCPs is desired but often not experienced by patients.^12^, ^13^, ^14^, ^15^, ^16^, ^17^ In Germany, PCC and SDM have been integrated into many clinical guidelines, curricula of most medical schools and health policies like the ‘Patients' Rights Act’ and the German National Cancer Plan (‘Nationaler Krebsplan’).^18^, ^19^, ^20^ However, PCC and SDM are still not fully implemented in routine cancer care, and studies on the effectiveness and implementation of PC communication and SDM are necessary to fill this gap.^5^, ^18^ In Germany, two larger‐scale studies investigating the implementation of SDM in routine cancer care by multiple strategies have recently been conducted (SHARE TO CARE and PREPARED).^21^, ^22^ While the SHARE TO CARE study reported encouraging findings,^22^ outcome evaluations of the PREPARED trial revealed no statistically significant intervention effects on the primary outcome of SDM as experienced by patients and on most secondary outcomes, including observer‐rated SDM in clinical encounters.^21^ However, within the PREPARED trial, it was not assessed whether the implementation programme might have had an impact on the general PC communication skills of physicians. The implementation programme consisted of six implementation strategies. Effects of the SDM implementation programme on PC communication of physicians can be expected since PC communication training and coaching (with a focus on SDM) were conducted within the trial and specifically addressed HCPs' PC behaviour.^21^

Thus, the aim of the present secondary analysis of data from the PREPARED trial was to assess whether the SDM implementation programme had effects on PC communication in clinical encounters in routine cancer care.

## 2 METHODS

### 2.1 Study design

This quantitative study is a secondary analysis of data gathered between March 2018 and October 2020 as part of the outcome evaluation of the cluster‐randomised SDM implementation trial PREPARED.^21^ The data used here included audio recordings before and after the roll‐out of the SDM implementation programme within the PREPARED trial.^21^, ^23^

### 2.2 Overview of the underlying SDM implementation trial PREPARED

The PREPARED trial aimed to increase SDM adoption through a comprehensive implementation programme including the analysis of influencing factors.^23^ This cluster‐randomised PREPARED trial, using a stepped wedge design, took place across three departments of the comprehensive cancer centre at the University Medical Center Hamburg‐Eppendorf (UKE).^24^, ^25^ Those departments were selected based on the expressed interest of the heads of the departments to implement SDM. They provided consent to study participation before randomisation. The three departments were randomised by a study team member (L. K.) using computer‐generated random numbers. Another study team member (I. S.) enroled clusters before randomisation by concealing the allocation sequence. Blinding of HCPs within departments was not feasible due to the nature of the implementation programme, patients were blinded for condition. Within this stepped wedge design, Department 1 switched from the control condition to the intervention condition first, followed by Departments 2 and 3 (see Supporting Information S1: File 1 for the trial design). It is important to note that the pool of eligible physicians for study participation fluctuated between measurement waves due to rotations within departments (a standard procedure in academic hospitals), as well as physicians departing or newly joining the department. The SDM implementation programme comprised six strategies at three levels: (1) The *HCP level* involved interdisciplinary training sessions for physicians and nurses using a train‐the‐trainer approach.^26^, ^27^ A group of multipliers underwent an intensive 5‐h training on PC communication skills with a special focus on SDM. Those training sessions included structure and aspects of a PC consultation, raising awareness for patients' needs and the patients' perspective, information on risk communication and tools for increasing PC, as well as training on applying the SDM ‘Three Talks Model’ in a clinical encounter.^6^ All other physicians and nurses participated in 1‐h interdisciplinary trainings, which were conducted by trained multipliers together with a study team member (A. L., P. H., I. S.). Compared to the 5‐h training, those shorter training programmes focused on raising awareness for the patients' perspective, applying SDM and the ‘Three Talks Model’ as well as PC interventions like decision aids.^6^ Additionally, physicians received two individual coaching sessions with feedback on PC communication (regarding PC structure of the consultation, and general as well as risk communication skills) and application of aspects of the SDM ‘Three Talks Model’ in observed encounters. (2) The *patient level* included posters and postcards of the patient empowerment strategy ‘Ask 3 Questions’,^28^ patient information material and a generic decision aid. (3) The *department level* activities involved integrating SDM into quality management documents and organising meetings with stakeholders to discuss multidisciplinary team meetings (tumour boards).

Further details on the design, methodology, outcome and process evaluation of the PREPARED trial are reported elsewhere.^21^, ^23^, ^29^

### 2.3 Outcome measure

In this study, the communication skills of participating physicians were analysed via the observer‐based instrument Four Habits Coding Scheme (4HCS).^30^, ^31^ This instrument has already been used in past studies to investigate the uptake of PC communication.^32^, ^33^, ^34^ Many translations and cultural adaptations of the 4HCS instruments exist,^35^, ^36^ including a validated German version.^31^ The 4HCS consists of 23 items, assigned to four habits: (1) the habit ‘invest in the beginning’ includes six items on building a relationship, evaluating patients' concerns, and planning the visit; (2) the habit ‘elicit the patients' perspective’ includes three items on the evaluation of the patients' feelings and understanding of their individual situation as well as how the illness affects their life; (3) the habit ‘demonstrate empathy’ includes four items on accepting the patients' emotions, providing empathy both verbally and nonverbally, and reflecting on own emotions and (4) the habit ‘invest in the end’ includes 10 items on providing information about the diagnosis, involving the patient in decisions regarding treatment, and appropriately finishing the consultation.^30^, ^31^ Items can be rated on a Likert scale from 1 ‘not effective at all’ to 5 ‘very effective’. In this study, the item ‘shows appropriate nonverbal behaviour’ of the third habit could not be rated since only audio recordings of the consultations were available. It is possible to calculate a score for each habit as well as an overall score, whereby a higher score indicates better PC communication skills.^30^, ^31^ For our data, overall scores between 22 and 110 were possible. Hence, for each of the four habits, the possible score ranges were: 6–30 for the habit ‘invest in the beginning’, 3–15 for the habits ‘elicit the patients' perspective’ and ‘demonstrate empathy’, and 10–50 for the habit ‘invest in the end’.

Inter‐rater reliability of the 4HCS was high in the original study adapting the measure into German.^31^ In the present analysis, inter‐rater reliability, type ‘ICC2,k’, according to the classification of Shrout and Fleiss^37^ was 0.548 (95% confidence interval: 0.370–0.675) for the habit ‘invest in the beginning’, 0.513 (0.165–0.706) for the habit ‘elicit the patients perspective’, 0.578 (0.292–0.736) for the habit ‘demonstrate empathy’, 0.782 (0.632–0.863) for the habit ‘invest in the end’, and 0.603 (0.30–0.760) for the overall 4HCS score.

### 2.4 Data collection

All physicians working in outpatient clinics of the three participating departments of the PREPARED trial were informed about the study and the data collection in encounters via e‐mail and in regular staff meetings. During the measurement waves of the outcome evaluation of the trial (see Supporting Information S1: File 1), members of the study team (A. L., P. H., W. F., J. Z., see Acknowledgements) visited the outpatient departments on different weekdays. They asked physicians offering consultation hours at those times to allow taking audio recordings of some of their oncological encounters. When the respective physician gave their approval, the study team informed the patient about the PREPARED trial, data collection and data handling and sought informed consent for the audio recordings. Due to the design of the PREPARED study and rotation of physicians within the departments, the inclusion of physicians, who did not take part in training and/or coaching, was possible. Patient inclusion criteria comprised being at least 18 years old, having an oncological disease and possessing adequate proficiency in the German language. After patients gave written informed consent, the study team member started the audio recording and left the room. After the encounter, patients were asked to fill out a short survey, including demographic questions and the Control Preference Scale (CPS).^38^ The CPS was assessed in its original version measuring preferred participation in decision‐making and in an adapted version measuring experienced participation in the previous encounter. Demographic data from physicians or data about participation in training and coaching or other implementation strategies within the PREPARED study were not collected. The aim was to record at least 12 audio recordings per department in each of the four measurement waves of the outcome evaluation.^23^ Per design, the allocation of audio recording per department to the intervention and control condition varied.

In audio files, sections that enabled identification of individuals (e.g., names of people and hospitals, dates of encounters) were anonymized using the software Audacity (Audacity Team) and transcribed verbatim using the software f4. To evaluate physicians' PC communication skills in the audio‐recorded encounters, transcripts of audio recordings were independently rated by two raters (L. M. and either A. K., H. S. or S. Z.) via the 4HCS. All raters participated in a 5‐h rater training beforehand. This training was conducted by I. S., who is an expert in the use of the 4HCS rating scale and has led its translation to German, its cultural adaptation and validation.^31^ According to the original 4HCS coding manual, the raters were instructed to preferably use the scores 1, 3 and 5 and use the scores 2 or 4 as rarely as possible and only if they have the impression that the physician's behaviour fell in between.^39^ This procedure increases variance and reduces floor and ceiling effects. Raters were blinded regarding the identity of the physician, name of the department, date of the audio recording and whether the consultation took place before or after the implementation of the SDM programme.

### 2.5 Data analysis

Demographic data of patients, characteristics of the audio recordings and results of the 4HCS ratings were entered into SPSS Statistics 27 (IBM Corp.). Descriptive statistics (frequencies, mean scores, standard deviations) were calculated for demographic data of the patients (e.g., age, gender, education), the CPS,^38^ characteristics of the audio recordings (e.g., number of audio recordings per department, mean duration) and for the overall 4HCS scores and each individual habit. Differences between the control (preimplementation) and intervention (postimplementation) conditions were evaluated via the Pearson *χ* ^2^ test for nominal data (department, patients' gender, first language and occupational status). Differences between control and intervention conditions for ordinal data (formal education, CPS scale) were evaluated via the Mann–Whitney‐*U*‐test. Differences between control and intervention conditions for metric data (patients' age, number of patients invited and included, duration of audio recordings, total and individual habit scores) were calculated via *t*‐test for independent samples. Differences between the three departments regarding the duration of the audio recordings were calculated via analysis of variance.

To evaluate the effect of the SDM implementation programme on communication skills, a linear mixed model was used.^40^, ^41^ Department was used as a random effect, the condition (control and intervention) as a fixed effect and the data collection period as a covariate. Statistically significant differences in demographic data between the intervention and the control condition were included as fixed effects to adjust for their influence on the outcome.

## 3 RESULTS

### 3.1 Characteristics of the audio recordings

Across all departments, 169 patients were asked to take part in audio recordings. patients refused to participate because of no specified reason (*n* = 11), emotional distress (*n* = 8), having already participated (*n* = 2), no interest in study participation (*n* = 1) or too much expected effort (*n* = 1). From the 146 included audio recordings of clinical encounters, 74 audio recordings were collected during the control condition (before implementation) and 72 during the intervention condition (after implementation). The total number of audio recordings was comparable for each department and the aim of at least 12 audio recordings per department and assessment phase could be reached. Forty‐eight audio recordings were collected in Departments 1 and 2 (each 32.9%), and 50 audio recordings were collected in Department 3 (34.2%). The mean duration of audio recordings over all departments was 18.76 min with a range between 3 and 49 min. Details on the characteristics of audio recordings are displayed in Table 1.

**Table 1:** Patient recruitment and encounter duration.

		Control condition (*n* = 74)				Intervention condition (*n* = 72)				Total
		Dep 1	Dep 2	Dep 3	Total	Dep 1	Dep 2	Dep 3	Total	
*Patient recruitment*										
Patients invited	*n* (%)	15 (16.9%)	29 (32.6%)	45 (50.6%)	**89 (100%)**	37 (46.3%)	30 (37.5%)	13 (16.3%)	**80 (100%)**	**169**
Patients included	*n* (%)	12 (16.2%)	24 (32.4%)	38 (51.3%)	**74 (100%)**	36 (50.0%)	24 (33.3%)	12 (16.6%)	**72 (100%)**	**146**
*Duration of clinical encounters (in min)*										
Mean (SD)		15.17 (6.60)	25.12 (8.42)	17.08 (9.06)	**19.38 (9.33)**	12.36 (7.96)	24.38 (9.45)	22.92 (10.65)	**18.13 (10.57)**	**18.76 (9.94)**
Range		6–26	7–41	4–45	**4–45**	3–40	9–49	10–45	**3–49**	**3–49**

Abbreviations: Dep, department; *n*, number of observations; SD, standard deviation.

### 3.2 Characteristics of the patient sample

Nearly 70.3% of the patients in the control condition and 44.4% of the patients in the intervention condition were female. This difference was due to Department 3 almost only treating female patients. The mean age of participants was 57.1 years (control condition) and 59.5 years (intervention condition). Most patients' first language was German (control condition: 78.4%, intervention condition: 77.8%). Additionally, the largest portion of participating patients had medium‐level formal education (control condition: 32.4%, intervention condition: 30.1%) and were (self‐)employed (control condition: 37.8%, intervention condition: 37.5%). In the CPS, most patients preferred a shared decision together with their physician in the past encounter (control condition: 52.7%, intervention condition: 69.4%), and most patients experienced SDM in this encounter (control condition: 68.9%, intervention condition: 69.4%). However, the proportion of missing data for those items was high. All above‐mentioned group differences were not statistically significant except for a statistically significant difference between groups regarding gender (*χ* ^2^ = 8.411, *df* = 1, *p* < .01). All details on sample characteristics can be found in Table 2.

**Table 2:** Demographic data of participants and results of CPS.

	Control condition (*n* = 74)		Intervention condition (*n* = 72)		Total (*n* = 146)		Statistical comparison of control and intervention condition
	*n*	%	*n*	%	*n*	%	
Gender, *n* (%)							
Female	52	70.3	32	44.4	**84**	**57.7**	*χ* ^2^ = 8.41, *df* = 1, *p* = .004
Male	22	29.7	37	51.4	**59**	**40.4**	
Missing	0		3	4.2	**3**	**2**	
Age							
Years, mean (SD)	57.1	13.8	59.5	15.8	**58.3**	**14.8**	*t* = −0.953, *df* = 132, *p* = .171
Range	27–84		21–92		**21–92**		
First language, *n* (%)							
German	58	78.4	56	77.8	**114**	**78.1**	*χ* ^2^ = 0.032, *df* = 1, *p* = .858
Others	8	10.8	7	9.7	**15**	**10.3**	
Missing	8	10.8	9	12.5	**17**	**11.6**	
Formal education, *n* (%)							
Low^a^	16	11.8	14	19.4	**30**	**20.5**	*U* = 1888.0, *Z* = −0.488, *p* = .626
Medium^b^	24	32.4	22	30.1	**46**	**31.5**	
High^c^	12	16.2	13	18.1	**25 25**	**17.1**	
Very high^d^	12	16.2	10	18.1	**20**	**17.1**	
Missing	10			13.9		**13.07**	
Occupational status,^e^ *n* (%)							
(Self‐)employed	28	37.8	27	37.5	**55**	**37.7**	*χ* ^2^ = 2.04, *df* = 1, *p* = .153
Retired	22	29.7	29	40.3	**51**	**34.9**	*χ* ^2^ = 0.001, *df* = 1, *p* = .982
Homemaker	7	9.5	5	6.9	**12 11**	**8.2**	*χ* ^2^ = 0.309, *df* = 1, *p* = .578
Other^f^	8	10.8	3	4.2	**22**	**0.7**	*χ* ^2^ = 9.0, *df* = 7, *p* = .253
Missing	11	14.9	11	15.3		**15.1**	
CPS—preference,^g^ *n* (%)							
Only patient	5	6.8	2	2.8	**7**	**4.8**	*U* = 1600.0, *Z* = −0.513, *p* = .608
Patient and physician together	39	52.7	50	69.4	**89**	**60.9**	
Only physician	0	0.0	1	1.4	**1**	**0.7**	
Missing	30	40.5	19	26.4	**49**	**33.6**	
CPS—experience,^h^ *n* (%)							
Only patient	4	5.4	7	9.7	**11**	**7.5**	*U* = 1058.0, *Z* = −1.642, *p* = .101
Patient and physician together	51	68.9	50	69.4	**101**	**69.2**	
Only physician	1	1.4	2	2.7	**3**	**2.1**	
Missing	18	24.3	13	18.1	**31**	**21.2**	

Abbreviations: CPS, Control Preference Scale; *df*, degree of freedom; *n*, number of observations; *p =* *p*‐value of the adjusted mean difference; SD, standard deviation.

^a^ Low = no formal degree or education after less than 10 years at school.

^b^ Intermediate = graduation after 10 or 11 years at school.

^c^ High = graduation after more than 11 years at school.

^d^ Very high = college or university degree.

^e^ Multiple choices possible.

^f^ Including trainee, student, sick leave, military service, unemployed.

^g^ Preference of patients regarding decision‐making: ‘Who should make the decision?’

^h^ Experience of patients with decision making: ‘Who made the decision?’

### 3.3 Intervention effects on communication skills as measured by the 4HCS

Table 3 provides descriptive statistics for the overall 4HCS score as well as the summed scores for each of the four habits in both groups. Overall mean values were 66.75 (SD = 9.97) for the control condition and 66.47 (SD = 11.28) for the intervention condition (on a scale between 22 and 110). For the first habit ‘invest in the beginning’ and the second habit ‘elicit the patient's perspective’, mean values were slightly higher for the intervention compared to the control condition. For the third habit ‘demonstrate empathy’ and the fourth habit ‘invest in the end’, mean values were slightly lower for the intervention compared to the control condition.

**Table 3:** Descriptive statistics for 4HCS scores for the control and intervention conditions.

	Control condition			Intervention condition		
	Mean	(SD)	Range	Mean	(SD)	Range
Overall score	66.75	(9.97)	44.00–93.00	66.47	(11.28)	45.05–91.67
Habit 1	15.78	(3.51)	9.00–25.00	17.52	(3.73)	6.60–24.50
Habit 2	7.61	(1.98)	3.50–14.00	8.28	(2.37)	3.50–14.00
Habit 3	8.1	(2.86)	4.00–14.50	6.87	(1.87)	4.00–12.50
Habit 4	35.13	(5.79)	23.00–46.00	33.7	(6.54)	20.00–47.50

*Note*: Habit 1 = invest in the beginning; Habit 2 = elicit the patients perspective; Habit 3 = demonstrate empathy; Habit 4 = invest in the end; possible scores between 22 and 110.

Abbreviations: 4HCS, Four Habits Coding Scheme; SD, standard deviation.

In the applied linear mixed model, we included gender as a fixed effect since a statistically significant difference could be found for this variable between intervention and control condition. No statistically significant effects could be found in the calculated mixed linear model for the overall 4HCS score as well as for the individual scores. The results of the linear mixed model calculations are displayed in Table 4.

**Table 4:** Results of mixed linear model calculations for overall 4HCS score as well as individual habit scores.

Estimated marginal means									Estimated values					
	Control condition				Intervention condition									
	Mean	SE	*df*	95% CI	Mean	SD	*df*	95% CI	ICC	aMD	SE	*df*	95% CI	*p*‐Value
Overall Score	63.91	3.41	21.33	56.82–71.00	65.44	1.79	5.59	60.96–69.92	.028	1.52	2.95	43.73	−4.43 to 7.48	.608
Habit 1	14.65	1.25	11.03	11.89–17.41	16.63	0.78	2.40	13.76–19.49	.093	1.98	1.00	80.43	−0.02 to 3.97	.052
Habit 2	6.84	0.76	16.79	5.823–8.45	8.05	0.45	3.63	6.73–9.36	.074	1.20	0.62	78.94	−0.04 to 2.45	.058
Habit 3	7.84	0.71	139	6.44–9.24	6.84	0.34	139	6.16–7.52	.000	0.99	0.63	139	−2.24 to 0.24	.113
Habit 4	34.80	2.17	15.97	30.19–39.41	33.75	1.29	3.42	29.91–37.59	.072	1.05	1.79	0.56	−4.61 to 2.51	.557

*Note*: *p* = *p*‐value of the adjusted mean difference (intervention effect estimate); Habit 1 = invest in the beginning; Habit 2 = elicit the patients perspective; Habit 3 = demonstrate empathy; Habit 4 = invest in the end; possible scores between 22 and 110.

Abbreviations: 4HCS, Four Habits Coding Scheme; aMD, adjusted mean difference; CI, confidence interval; *df*, degree of freedom; ICC, intraclass correlation coefficient; *n*, number of observations; SE, standard error.

## 4 DISCUSSION

The present study aimed to assess whether an SDM implementation programme for routine cancer care had effects on PC communication in clinical encounters assessed by the observer‐based 4HCS scale.^21^ We did not identify any statistically significant difference between the control and intervention conditions regarding the 4HCS total and individual habit scores. This means that we did not find effects of training and coaching conducted as part of the PREPARED trial on PC communication of physicians in clinical encounters.

In our study, overall 4HCS scores for the control condition (mean = 66.75, SD = 9.97, range = 44.00–93.00) are slightly higher compared to some studies on the effectiveness of training on PC communication skills, even though we excluded the item on nonverbal communication. Jensen et al.^32^, ^42^ found mean values of 60.3 (SD = 9.9) and 60.1 (SD = 14.4) in their baseline samples of physicians. Gulbrandsen et al.^43^ found a baseline rate of 58.8 (SD = 9.8) in their participating physicians. Higher values (mean = 76.44, SD = 12.34) could be found in the French adaptation study analysing data of consultations between medical students and simulation patients.^36^ Thus, in accordance with other studies on the effectiveness of training on PC communication skills, an improvement of our baseline 4HCS values could have been expected.^32^, ^34^, ^44^ In a recent study by Amsalem et al.^34^ evaluating a 1‐day communication skills training in clinical psychiatry using simulated patients, the 4HCS total scores significantly improved from pre to post‐training. Jensen et al.^32^ showed a statistically significant increase in the 4HCS total score after a 20‐h communication skills training. Compared to our training, training conducted within those studies was of longer duration and/or was developed based on the Four Habits model.^32^, ^34^, ^45^ Furthermore, those studies evaluated the effectiveness of the training programme on the level of individual participants, that is only data from physicians engaging in communication skills training were collected. In contrast, the PREPARED trial was designed as an implementation study in which we evaluated the SDM implementation programme (which included training and coaching) at the department level. That is, we collected data throughout the department irrespective of which physicians had been exposed to the training or coaching. When examining the content of the training and coaching sessions alongside the items of the 4HCS, it becomes apparent that there is a convergence in addressing certain aspects of PC communication, such as organising a consultation focused on the patients and understanding the patients' viewpoint. However, certain elements of the 4HCS (such as assessing the patients' emotions, acknowledging their feelings, demonstrating empathy and reflecting on one's own emotions) were not specifically emphasised in the PREPARED trial. This omission somewhat hindered the suitability of employing the 4HCS scale in this context. Further reasons for the lack of effect in our data might be due to the limited reach of the SDM implementation programme and several barriers, which had been evaluated in a comprehensive process evaluation of the PREPARED trial.^29^ Those barriers could be found on five levels: (1) characteristics of the intervention (e.g., translation into practice, adaptability), (2) the individual level (e.g., personal relevance, individual motivation to change), (3) the level of the inner setting (e.g., limited time and resources, leadership engagement), (4) the level of the outer setting (e.g., culture of healthcare delivery, payment models) and (5) the implementation process (e.g., participation during working hours, integration into existing structures).^21^, ^29^ One important reason for the lack of effect found in this study might be the limited reach of the training. Across all departments, only 52% of the eligible physicians participated in the training.^21^ One reason might be the high fluctuation of staff and rotations of physicians between departments. Furthermore, the duration of the team training was rather short. Instead of the a priori planned 2‐h team training, the mean duration of the conducted team training was 50 min due to working routines within the departments.^21^ Previous studies found that the duration of communication skills training affects observed communication skills. Barth and Lannen^46^ found that their communication skills training had effects on the communication skills of physicians. Those effects were higher in participants attending more intensive training. However, they argue that feasibility and economic aspects have to be considered when planning training.^46^ Another explanation might be, that the PREPARED intervention did not sufficiently change the attitudes of physicians regarding PC communication (and SDM).^29^ This might have prevented a change in the physician's behaviour.^47^ Cognitive rigidity and reduced openness for implementing a new habit as well as fear of not behaving in the patients' interest can reduce changing a behaviour.^48^, ^49^ The PREPARED process evaluation highlighted several barriers that might have influenced physicians' willingness to adopt PC communication skills or SDM in their encounters.^29^ Those include limited time and a set of other priorities, which are known barriers to adopting a new behaviour.^49^, ^50^ This illustrates the need to further evaluate and address influencing factors on implementation, specifically openness and motivation of HCPs to implement a new behaviour like PC communication or SDM.

## 5 STRENGTHS AND LIMITATIONS

Strengths of this study include the large number of recordings from nearly unselected patients from three cancer care departments and the data collection within routine cancer care. We furthermore safeguarded data quality by conducting training on 4HCS rating for all involved raters and blinding raters regarding the identity of the physician, departments, date of recruitment and implementation status.

However, this study shows several limitations which primarily result from the study design as a secondary analysis. First, the PREPARED trial expected implementation effects on the level of the departments (independently from training and coaching participation of single HCPs). Thus, data on physicians' participation in training and coaching or their participation or use of other implementation strategies was not collected and could not inform the interpretation of the results of the study at hand. Second, the effectiveness of the SDM training and coaching on SDM uptake or PC communication was not evaluated before rolling out in the PREPARED trial. Thus, we do not know if the lack of effect found in this study might be due to the lack of effect of training and coaching on PC communication uptake or the limited reach of the intervention in the target population. To counteract these limitations, the effects of SDM and PC communication training on PC communication in clinical encounters should be evaluated in a primary trial first. Third, moderate interrater reliability might have reduced the probability of identifying existing intervention effects in this study. Fourth, data collection in routine care was already mentioned as a strength of this study, but the resulting clinical heterogeneity might also be a limitation of our study. Clinical encounters were selected somewhat indiscriminately, displaying a notable variability in both duration and topics discussed among patients and physicians. Some of the recorded encounters were rather short. Also Krupat et al.^30^ reported an association between the duration of encounters and 4HCS ratings. In their study, high‐level PC communication skills could only be found in longer encounters.^30^ There might be several other influencing factors, which could not be controlled or changed within a real‐world setting. Those include the severe acute respiratory syndrome coronavirus 2 pandemic, which caused a delay in the last measurement wave of the PREPARED trial and might have influenced routine healthcare practices.^29^ However, there were no other major influencing changes on the level of the healthcare system during the roll‐out of the PREPARED trial. Furthermore, the generalisability of our results should be discussed with caution since the PREPARED trial was conducted in only three departments within a single cancer centre. Fifth, the rating was challenging for some of the topics discussed by the physicians and the patients. The first item ‘[the physicians] shows familiarity with the patient’ could often not be rated, since it was the first appointment of the patient with the respective physician. Some encounters focused only on organisational issues like planning the next appointment, without discussing a diagnosis or treatment options. For example, the 4HCS item ‘[the physician] helps to identify emotions’ was not applicable in those cases. Finally, this study included data from patients, but patients and the public were not engaged in the research process or the development of training and coaching. By involving especially patients in the identification of research priorities, development of interventions as well as the evaluation and dissemination process, the relevance of research and the development of feasible and accepted interventions that fit the needs of the target group can be improved.^51^ Thus, patient and public involvement should be integrated into future research in this field.

## 6 CONCLUSION

In this secondary analysis, we did not find a statistically significant increase in the uptake of PC communication in clinical encounters after rolling out an SDM implementation training in three departments of a German comprehensive cancer centre. It is unlikely that further studies on the net effect of training interventions on PC communication would lead to a substantial knowledge gain. Instead, future investigations should focus on understanding how and under which circumstances these interventions work by examining possible predictors, mediators, and moderators of the effect, such as attitudes, readiness to change and contextual factors. An exploration of the working mechanisms of training could largely enhance our possibilities to implement and improve PC communication in cancer care.

## AUTHOR CONTRIBUTIONS

**Anja Lindig**: Writing—original draft; project administration; data curation; writing—review and editing. **Lotta Mannagottera**: Writing—original draft; writing—review and editing; formal analysis. **Pola Hahlweg**: Data curation; writing—review and editing; project administration; supervision. **Hannah Sigl**: Formal analysis; writing—review and editing. **Anne Klimesch**: Formal analysis; writing—review and editing. **Stefan Zeh**: Formal analysis; writing—review and editing. **Levente Kriston**: Conceptualisation; formal analysis; methodology; supervision; writing—review and editing. **Isabelle Scholl**: Conceptualisation; methodology; supervision; project administration; writing—review and editing; funding acquisition.

## CONFLICT OF INTEREST STATEMENT

Pola Hahlweg and Isabelle Scholl declare that they currently are (Pola Hahlweg) or have been (Isabelle Scholl) members of the executive board of the International Shared Decision Making Society, which has the mission to foster SDM implementation. Pola Hahlweg and Isabelle Scholl have no further competing interests. All other authors declare no conflict of interest.

## ETHICS STATEMENT

This study was carried out according to the latest version of the Helsinki Declaration of the World Medical Association, respected principles of good scientific practice and met standards of research ethics. The PREPARED trial was approved by the ethical review board of the Hamburg Chamber of Physicians (PV5368). Data protection and confidentiality requirements were met. Study participation was voluntary. All participants received information about the aims of the study, data collection and the use of collected data, and gave written informed consent before participation.

## Supporting information

Supporting information.

## Data Availability

The data that support the findings of this study are available on request from the corresponding author. The data are not publicly available due to privacy or ethical restrictions. Investigators who propose to use the data have to provide a methodologically sound proposal directed to the corresponding author. Signing a data use/sharing agreement will be necessary, and data security regulations both in Germany and in the country of the investigator who proposes to use the data must be complied with. Since single persons might still be identified in anonymized audio recordings (e.g., by voice), only anonymized transcripts of audio recordings are available. Quantitative data from patient surveys including variables that are necessary for replication of the analysis (without demographic and clinical data) are available.
