# Supplementary figures and images for: Effects of a shared decision‐making implementation programme on patient‐centred communication in oncology—Secondary analysis of a randomised controlled trial
Source: Health Expect. 2024 Mar 28;27(2):e14030. doi: 10.1111/hex.14030 (PMC10979048; doi:10.1111/hex.14030)

**Additional File 1: Stepped wedge study design of the PREPARED study**


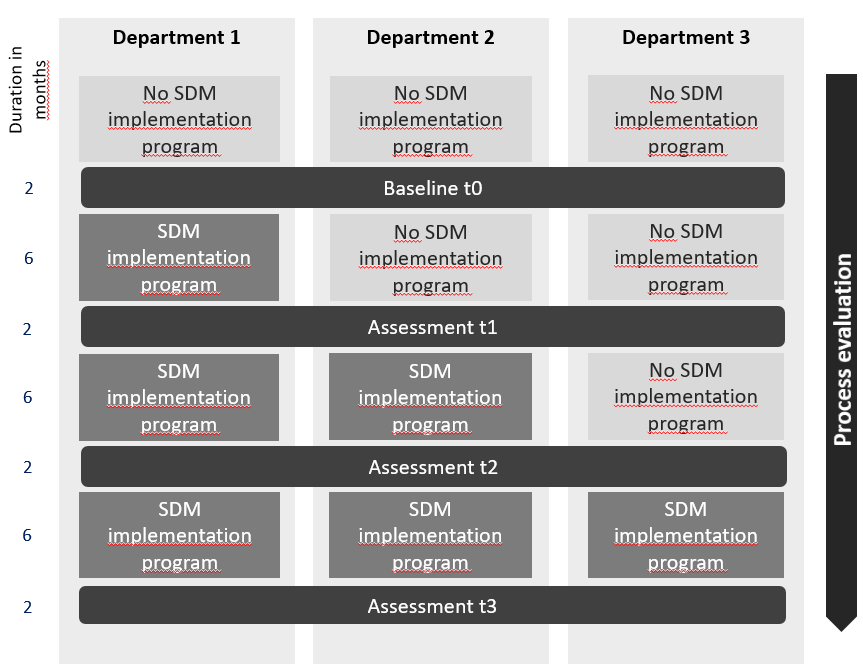

Supplement: Supplementary file 1 — Supporting information. [file HEX-27-e14030-s001.docx]
